# Novel spectrophotometric methods for concurrent assessment of duloxetine and avanafil in their binary mixture using derivative spectroscopy: greenness-blueness evaluation

**DOI:** 10.1186/s13065-025-01450-0

**Published:** 2025-04-02

**Authors:** Hadeer A. Elhamdy, Sayed M. Derayea, Khalid M. Badr El-Din, Mohamed Oraby

**Affiliations:** 1 Department of Pharmaceutical Analytical Chemistry, Faculty of Pharmacy, Sohag University, Sohag, 82524 Egypt; 2 Analytical Chemistry Department, Faculty of Pharmacy, Minia University, Minia, 61519 Egypt; 3 Pharmaceutical Chemistry Department, College of Pharmacy, Al-Esraa University, Baghdad, Iraq

**Keywords:** Duloxetine, Avanafil, Derivative spectra, Greenness evaluation

## Abstract

Antidepressant drugs, particularly selective serotonin reuptake inhibitors (SSRIs) and serotonin-norepinephrine reuptake inhibitors (SNRIs) are known to induce sexual dysfunction as a side effect. Duloxetine (DLX) and Avanafil (AVN) are simultaneously determined in their pure state and laboratory-prepared mixtures by two novel, environmentally friendly spectrophotometric methods. The first method was based on second order derivatives while the second method is based on first derivative dual-wavelength. In method I, the linearity range was found to be 0.5–12 µg/mL and 1–12 µg/mL with limit of detection 0.15 µg/mL and 0.27 µg/mL for DLX and AVN, respectively. In method II, the linearity range was found to be 1–10 µg/mL for both drugs with limit of detection 0.18 µg/mL and 0.21 µg/mL for DLX and AVN, respectively. The validation of these approaches meets the International Council for Harmonization’s (ICH) standards. Furthermore, three current ecological tools namely the Eco-Scale, GAPI, and AGREE were used to evaluate the proposed method’s greenness. The sustainability characteristics of the proposed method were also assessed using the Blue Applicability Grade Index (BAGI), a recently developed metric for assessing the practicality (blueness) of procedures.

**Supplementary Information:**

The online version contains supplementary material available at 10.1186/s13065-025-01450-0.

## Introduction

**Fig. 1:**
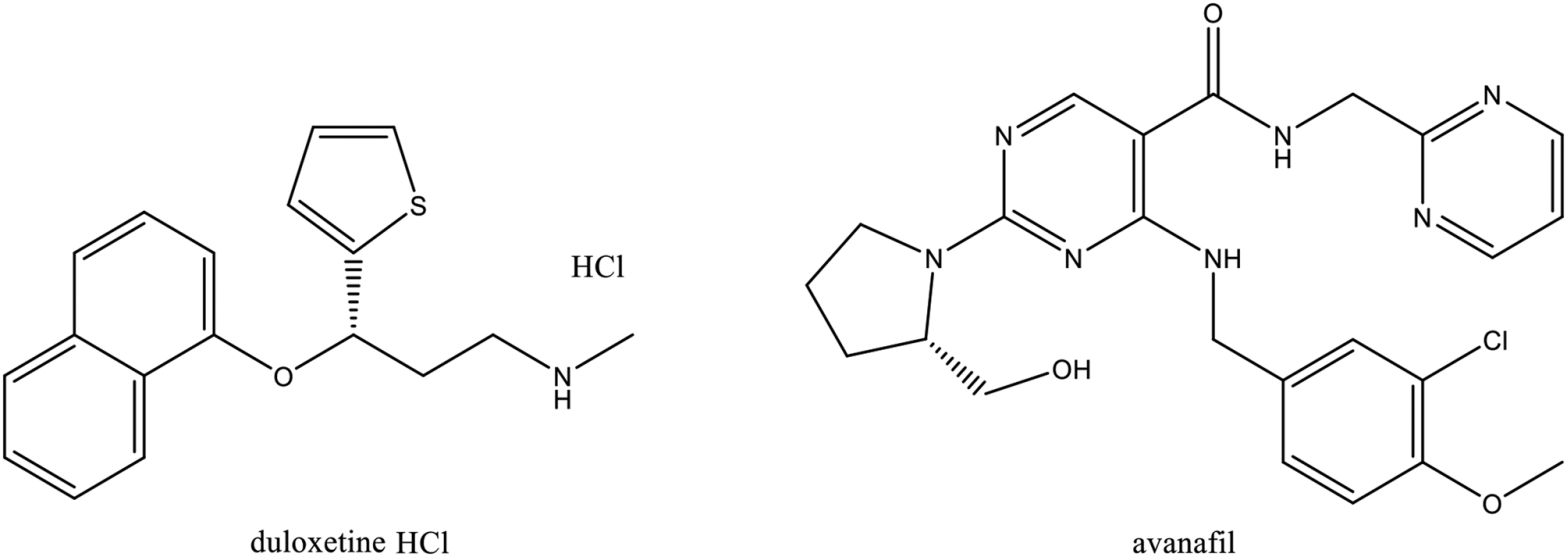
The chemical structures of the studied drugs

Duloxetine (DLX, Fig. 1) is known as 2(+)-(S)-N methyl-(gamma)-(1-naphthyloxy)-2 thiophen propylamine hydrochloride) [1]. With advantages like mood improvement, better sleep, decreased worry, and increased energy and appetite, DLX has been used to treat depressive disorders and anxiety [2]. DLX offers many advantages over antidepressant medications, such as improved safety, higher efficacy, tolerance, and fewer side effects. Additionally, it has a decreased affinity for brain receptors and dual inhibitory properties [3]. Numerous analytical techniques have been addressed in the literature of DLX including spectrophotometry [4–7], spectrofluorimetry [3, 8–10], TLC [1, 2, 11–13], electrochemical methods [14–17], gas chromatography [18], and HPLC [19–21].

The cGMP-specific type 5 phosphodiesterase (PDE5) is directly inhibited by avanafil (AVN), so erectile dysfunction is being treated with it [22]. Compared to other PDE5 inhibitors, avanafil has the advantage of starting to work significantly more quickly, easily absorbed and takes 30 to 45 min to reach its maximum concentration [23]. By blocking phosphodiesterase type-5, AVN mediates the antiproliferative and vasodilatory effects of endogenous nitric oxide, which is released by endothelial cells lining the arteries. This raises cGMP in the penile vasculature and contributes to a powerful erection. Blurred vision, abrupt hearing or vision loss, dyspnea, and fast heartbeat are all signs of AVN overdose. Despite all of these negative side effects, its use is still recommended over the other PDE-5 drugs. The chemical name for AVN (Fig. 1) is as follows: (S)-4-[(3-chloro-4-methoxybenzyl)amino].-2-[2-(hydroxymethyl)-1-pyrrolidinyl]-N-(2-pyrimidinylmethyl)-5-pyrimidinecarboxamide [24]. According to the literature review, numerous methods have been reported for the quantitative determination of AVN such as spectrophotometry [25, 26], spectrofluorimetry [23, 24, 27], HPLC [22, 28, 29], LC/MS [30–32], TLC [33], voltammetry [34–36] and capillary electrophoresis [37].

One adverse consequence of depression medications, specifically selective serotonin reuptake inhibitors (SSRIs) and serotonin norepinephrine reuptake inhibitors (SNRIs), is sexual dysfunction [38]. AVN is a preferable treatment for erectile dysfunction and other undesirable sexual adverse reactions. Therefore, the simultaneous assessment of DLX and AVN was the primary purpose of the present work. The proposed methods are simple so that it can be used for simultaneous measurement of DLX and AVN in their mixture accurately. As many physicians recommend AVN for the treatment of erectile dysfunction and other undesirable side effects of antidepressants including DLX, it is expected that such drugs combination maybe approved in the future.

There is a new method that has been published recently to measure two medications together and it explains the importance of giving both drugs to treat sexual dysfunction resulting from depression [39]. This study offers a new, accurate, selective, and green approach for determining the cited drugs in bulk and laboratory-prepared combinations in agreement with the ICH standards. There is no previously published UV spectrophotometric method for measuring DLX and AVN simultaneously, which makes the current work novel. This prompted the authors to develop a novel technique based on first and second derivative spectra for the concurrent determination of DLX and AVN.

## Experimental

### Instrumentation

A T80 double beam UV–VIS spectrophotometer (PG instruments, Leicestershire, UK) and UV Win software were used to make the spectrophotometric measurements. The measurements were made using centimeter-sized quartz cells. Double-distilled Aquatron water still A4000D (Cole-Parmer, Staffordshire, UK).

### Chemicals, standards, and samples

Mash Premiere Pharmaceuticals (Badr City, Cairo, Egypt) graciously supplied the DLX powder (99.6% purity), and Andalous Pharma (6th of October city, Cairo, Egypt) provided the AVN powder (99.9% purity). Cymbatex^®^ 30 mg (B.N. 2406920) (Eva pharma, Giza, Cairo, Egypt) and Erovanafile ^®^ 200 mg (B.N. 220346) (Andalous Pharma 6th of October City, Cairo, Egypt) were purchased from local market. HPLC grade methanol that are used were acquired from Merck in Darmstadt, Germany.

### Preparation of standard solution

A solution containing 1000 µg/mL of AVN and DLX was prepared by transferring 25 mg of each drug into 25-milliliter calibrated flasks and dissolved by methanol. Pipette 0.5 mL of the AVN stock solution and DLX into 10-mL flasks to form the working standard solution. Next, use methanol to adjust the concentration until it reaches 50 µg/mL. Various quantities of AVN and standard DLX were made using the same diluent. These solutions had been preserved in the fridge for future use.

### Procedure for pharmaceutical preparations

From 10 tablets of Erovanafile 200 mg that have been finely powdered, a precisely weighed quantity of tablet powder equivalent to 50 mg of AVN was put into a 50 mL volumetric flask. After thoroughly combining the contents of ten 30 mg Cymbatex^®^ capsules, a precise weight was used to transfer 50 mg of DLX of powder into the same flask. After that, the medications are extracted using methanol by sonicating for half an hour. Ultimately, the same solvent was used to raise the volume to 50 milliliters. After filtering the solution, the initial part of the filtrate was thrown away. The usual assay method was used to make five determinations for every concentration.

### General assay procedures

#### Method I (second derivative spectrophotometric method)

Employing the Second Order Derivative Method, Portions of the DLX and AVN standard stock solutions were carefully transferred inside separate 10 mL volumetric flasks, and then the methanol was carefully added. Every solution was subjected to scanning between 200 and 400 nm in the spectrum setting. Consequently, absorption spectra ranging from first to fourth order have been developed. For DLX and AVN analysis, two second-order derivative (scaling factor is 5 and Δλ = 5) spectra were selected because of their superior linearity and sensitivity. After the calibration curves were generated, the concentration of each drug in the combination of drugs was measured against the calibration curve in quantitation mode.

#### Method II (first derivative dual-wavelength method)

Methanol was used for completing the volumes after precisely transferring portions of the DLX and AVN standard solutions into 10 mL volumetric flasks. The first derivative spectra were produced after scanning the absorption spectra between 200 and 400 nm (scaling factor is 5 and Δλ = 5). The amplitude difference of the first derivative at 236 and 250 nm was plotted against the drug concentration in order to generate the calibration curve for DLX. While, the amplitude difference of the first derivative at 231.2 and 242.5 nm was plotted against the medication concentration for AVN.

## Result and discussion

**Fig. 2:**
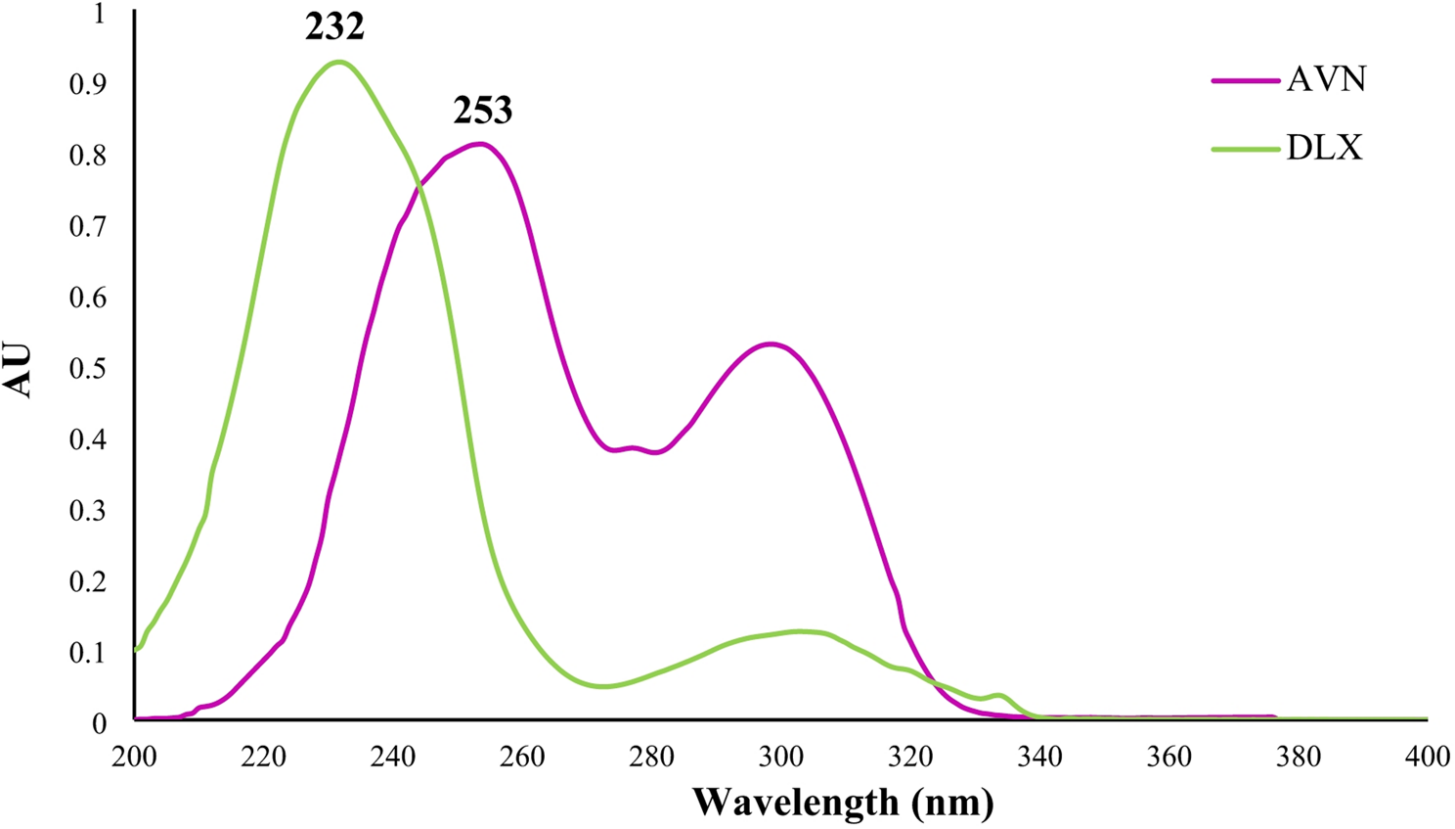
Absorption spectra of DLX and AVN (10 µg/mL) in methanol solvent

**Fig. 3:**
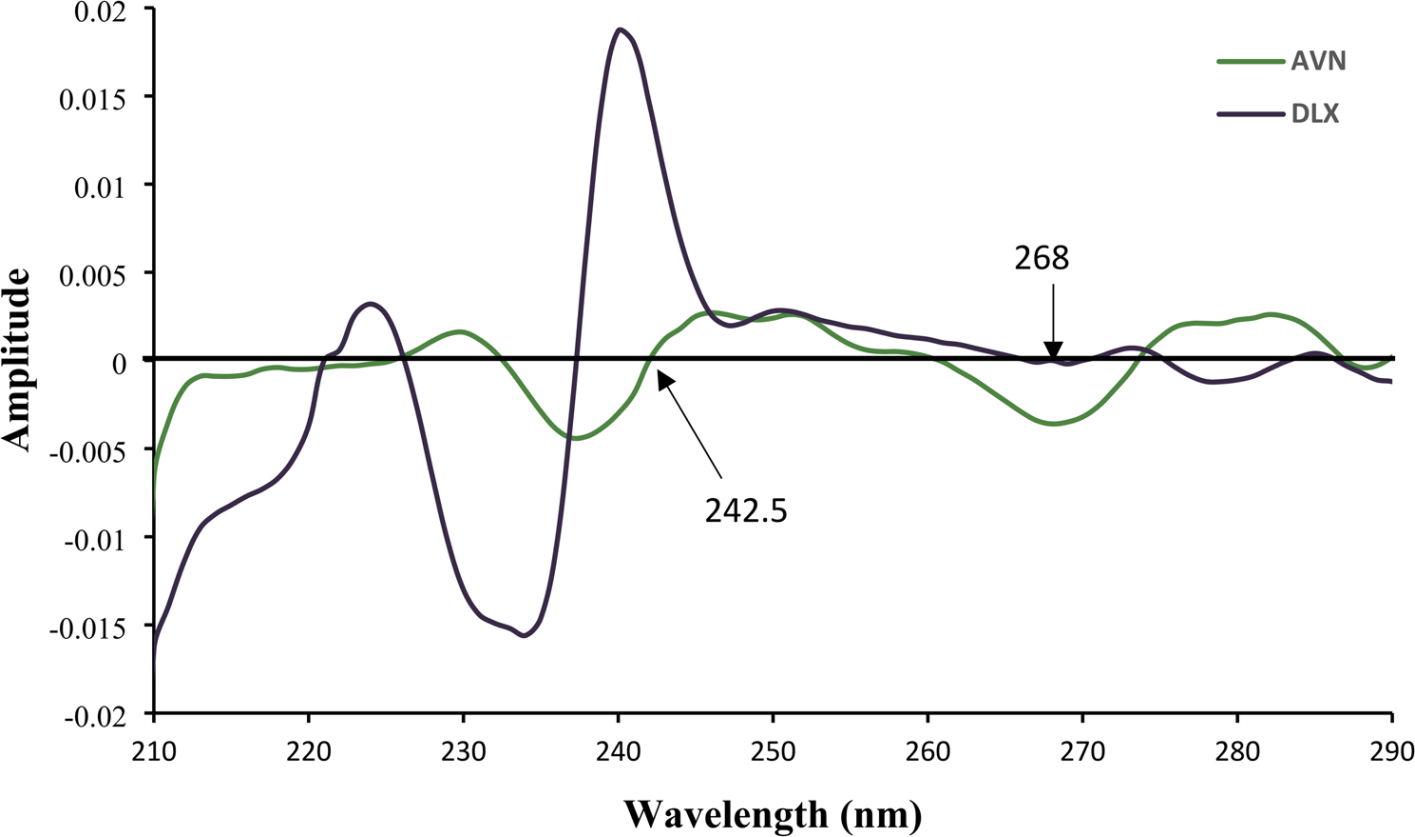
Second-derivative UV absorption spectra of DLX and AVN (6 µg/mL), showing zero crossing points at 268 for DLX and 242.5 nm for AVN

Considering DLX and AVN’s high UV absorption and solubility, methanol was subsequently chosen as the solvent for the current analytical techniques. Figure 2 displays the zero-order overlay UV spectra obtained for DLX and AVN (6 µg/mL each) which show that more than 90% of the spectra overlap, highlighting the difficulty of measuring these medications directly by UV absorption. To solve this overlap, derivative spectra were driven for both drugs, beginning from the zero-order spectra and progressing up to four orders. It was observed that, the second-order derivative spectra of both drugs identified specific regions for precise and linear detection of drugs. Because higher-order derivative spectra showed less linearity and sensitivity, second-order derivative spectra and first derivative dual wavelength method were selected for quantitative analysis. Second derivative spectra for AVN and DLX scanned in methanol are depicted in Fig. 3.

**Fig. 4:**
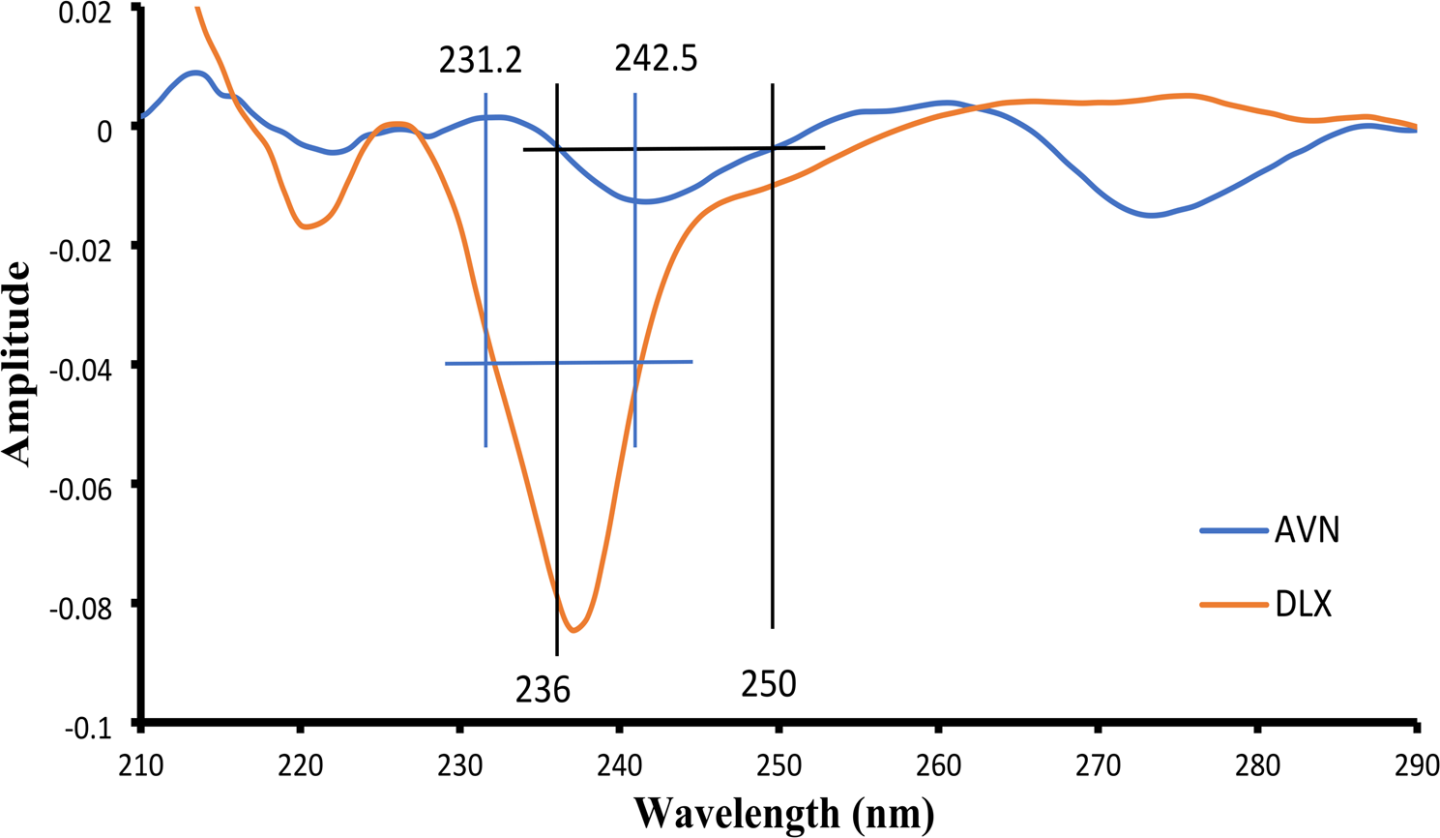
First-derivative of the UV absorption spectra of DLX and AVN (6 µg/mL), showing the dual wavelengths for each drug

For the first derivative Dual-wavelength method, the overlapped spectra of AVN and DLX at various concentrations demonstrated that AVN at various concentrations showed equal amplitude at 236 nm and 250 nm, at which, DLX showed a significant amplitude difference. Similarly, different DLX concentrations displayed similar amplitude at 231.2 nm and 242.5 nm, while AVN demonstrated a notable variation in amplitude. Hence, the wavelengths 236 nm and 250 nm were chosen for the estimation of AVN while 231.2 nm and 242.5 nm were chosen for the estimation of DLX based on the aforementioned facts, Fig. 4.

### The solvent choice regarding green ranking

**Fig. 5:**
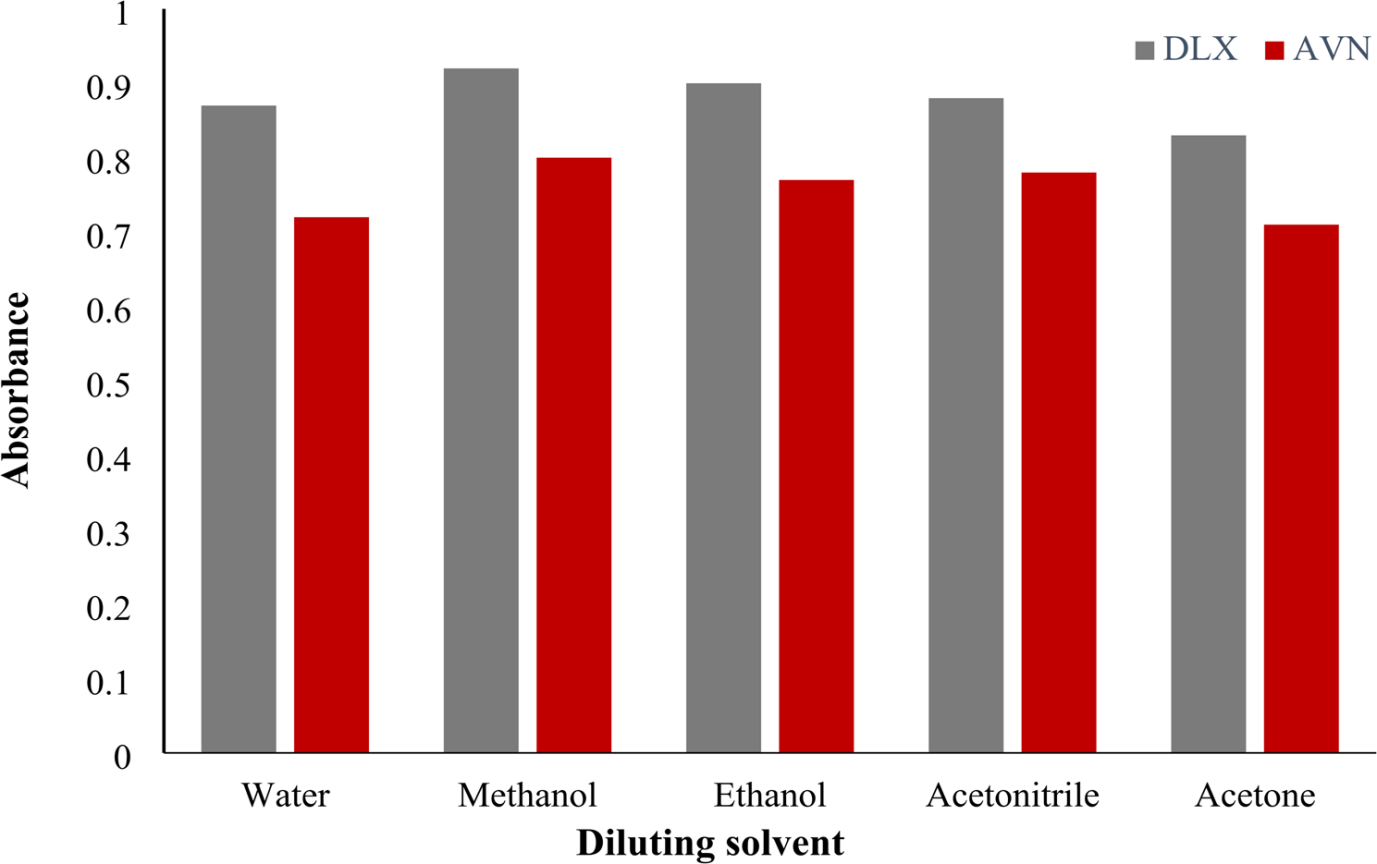
Effect of diluting solvent on absorbance of DLX and AVN (10 µg/mL)

Choosing a solvent is now strongly encouraged due to organic solvents’ detrimental effects on the environment and human health. Green analytical chemistry-related characteristics were used to evaluate the solvents and make the final selection [40]. The goal of the work strategy was to replace common organic solvents with safer substitutes which also diminishes the impact of hazardous solvents. Up till now, methanol has served as a viable organic solvent alternative (Fig. 5).

### Method validation

The approaches were verified in terms of variables, linearity range, accuracy, precision, LOQ, and LOD [41].

#### Linearity

The calibration graphs for determining DLX and AVN using second derivative method cover the concentration range of 0.5–12 µg/mL and 1–12 µg/mL, respectively. Meanwhile, the calibration curves for determination of DLX and AVN utilizing the dual-wavelength - first derivative method over the range of 1–10 µg/mL for both drugs. The high values of correlation coefficients supported the excellent linearity of the calibration curves. The analytical data including the slope and intercept of the calibration curves are summarized in Table 1.

**Table 1:** Statistical data of some analytical parameters of the proposed methods

Parameters	Method I (second derivative)		Method II (dual wavelength 1st derivative)	
	DLX	AVN	DLX	AVN
Linear range (µg/mL)	0.5–12	1–12	1–10	1–10
Slope ± SD	0.0033 ± 2.3E-05	0.0007 ± 7.83E-06	98.20 ± 0.89	9.44 ± 0.10
Intercept ± SD	0.0003 ± 0.00015	0.0003 ± 5.76E-05	-22.76 ± 5.43	11.63 ± 0.61
r *	0.9999	0.9998	0.9998	0.9998
Number of determinations	5	5	5	5
LOQ (µg/mL)	0.15	0.27	0.17	0.22
LOQ (µg/mL)	0.45	0.82	0.51	0.68

* r is correlation coefficient

#### LOQ and LOD

Based on the calibration graph’s slope and intercept standard deviation, the LOD and LOQ values were calculated. For the second derivative method, the LOD and LOQ values were found to be 0.15 and 0.27 µg/mL for DLX and 0.45 and 0.82 µg/mL for AVN, respectively. While the LOD and LOQ values for the dual wavelength method, were 0.18, 0.21 µg/mL for DLX and 0.55, 0.65 µg/mL for AVN, respectively. These values indicate a high sensitivity of the proposed methods (Table 1). The ICH equations: LOD = 3.3SD/b and LOQ = 10SD/b were utilized to calculate the LOD and LOQ, (b = slope and SD = standard deviation of the intercept).

#### Accuracy

The average % recovery was calculated for each of five different levels of drug concentration (2, 4, 6, 8, and 10 µg/mL) for both medications in triplicates for the two methods to assess the accuracy of the resulting approaches. The great reliability of the established approaches was proved by the accepted percent of recovery, which is listed in Table 2.

**Table 2:** Evaluation of the accuracy of the proposed methods for the determination of the investigated drugs

Drug	Conc. (µg/mL)	Method I		Method II	
		Amount found (µg/mL)	% Recovery ^a^ ± SD	Amount found (µg/mL)	% Recovery ^a^ ± SD
**DLX**	2	1.96	98.09 ± 1.74	1.99	99.34 ± 1.79
	4	3.99	99.79 ± 1.74	4.00	99.99 ± 1.67
	6	6.03	100.56 ± 1.62	6.05	100.78 ± 0.85
	8	8.06	100.81 ± 1.85	8.13	101.68 ± 1.21
	10	10.00	100.01 ± 1.16	10.06	100.61 ± 0.89
**AVN**	2	2.04	101.92 ± 1.75	2.02	100.86 ± 1.86
	4	3.99	99.79 ± 1.34	4.02	100.41 ± 1.46
	6	5.93	98.90 ± 1.16	5.99	99.85 ± 1.35
	8	8. **µg/mL** 09	101.17 ± 0.89	7.93	99.17 ± 1.75
	10	9.94	99.41 ± 1.34	9.86	98.58 ± 1.62

^a^ Mean of three determinations

#### Precision

For the objective of repeatability and intermediate precision, three-drug levels of concentration (2, 6, and 10 µg/mL) were determined in triplicates for both medications using the two methods throughout a single day, and on three consecutive days. The %RSD was used to measure the precision of the approaches that were developed. Table 3 lists the results of the suggested approaches, and their high precision was indicated by their small %RSD values.

**Table 3:** Evaluation of intra-day and inter-day precisions for the determination of the investigated drugs with the proposed methods

Method	Drug	Conc. (µg/mL)	Intra-day precision		Inter-day precision	
			% recovery ± SD ^a^	%RSD	% recovery ± SD ^a^	%RSD
**Method I**	**DLX**	2	99.09 ± 1.74	1.78	99.80 ± 1.86	1.86
		6	100.56 ± 1.62	1.61	100.42 ± 1.36	1.35
		10	100.01 ± 1.16	1.16	99.45 ± 1.30	1.31
	**AVN**	2	101.92 ± 1.75	1.72	101.84 ± 1.80	1.77
		6	98.90 ± 1.16	1.17	98.25 ± 1.43	1.46
		10	99.41 ± 1.34	1.34	100.06 ± 1.12	1.12
**Method II**	**DLX**	2	99.34 ± 1.79	1.80	99.48 ± 1.93	1.94
		6	100.78 ± 0.85	0.84	100.57 ± 1.37	1.36
		10	100.61 ± 0.89	0.88	100.48 ± 0.95	0.95
	**AVN**	2	100.86 ± 1.86	1.84	100.39 ± 1.34	1.33
		6	99.85 ± 1.35	1.35	100.44 ± 1.59	1.58
		10	98.58 ± 1.62	1.64	98.76 ± 1.32	1.34

^**a**^ Mean of three determinations

#### Robustness

It was obvious that the procedure was extremely simple, and the sole experimental factor that could be changed to test the method’s robustness was the measurement wavelength. Changing the excitation wavelength by ± 2 nm had no meaningful influence on percentage recovery. As a result, it is possible to conclude that the existing methodology is quite robust.

### Application to pharmaceutical dosage forms

Using the previously mentioned procedures, it was possible to successfully determine DLX and AVN in the pharmaceutical dosage forms. A statistical analysis was conducted to compare the outcomes of the proposed approaches with those attained by reported method [39]. To compare the two approaches statistically, the t-test and F-test were used. The F- and t-test calculated values were less than their 95% confidence level reference values, indicating that there were no discernible variations in accuracy and precision between the recommended and published methods (Table 4).

**Table 4:** Comparison between the results of the Estimation of DLX and AVN in their pharmaceutical preparations using the proposed and reported methods

Parameter	Method I		Method II		Reported method	
	DLX	AVN	DLX	AVN	DLX	AVN
**Mean % recovery** ^**a**^	100.37	99.59	100.27	100.61	101.24	99.63
**SD**	0.74	1.32	1.20	1.44	0.63	0.92
**t-test** ^**b**^	2.00	0.06	1.60	1.28		
**F-value** ^**b**^	1.38	2.06	3.63	2.45		

^a^ Mean of five measurements

^b^ Tabulated value at 95% confidence limit, F = 6.388 and t = 2.306

### Greenness assessment

**Fig. 6:**
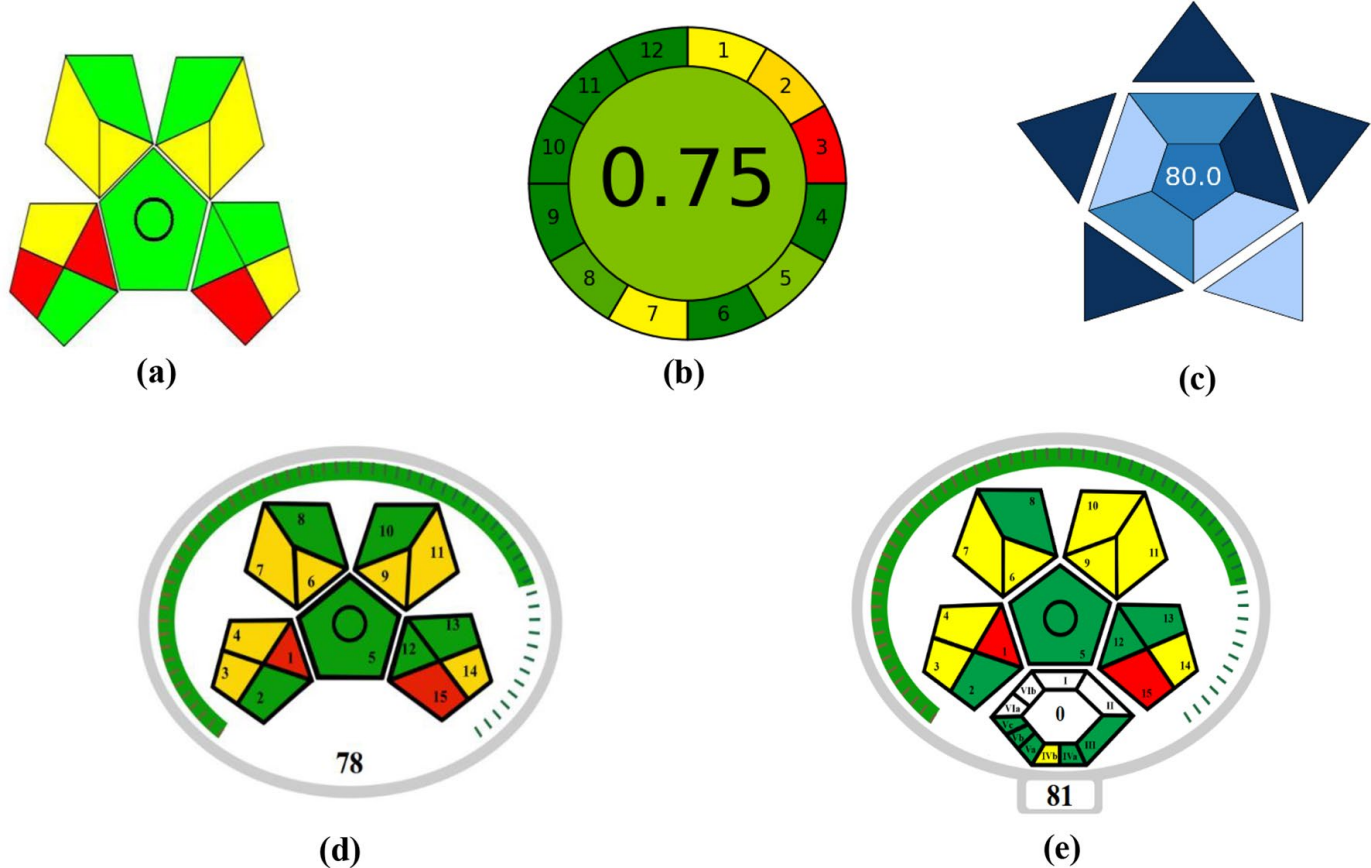
Evaluation of the greenness using GAPI (**a**), AGREE (**b**) metrics, the blueness using BAGI (**c**) MoGAPI (**d**) and complex MoGAPI (**e**) of the proposed spectrophotometric method

The green analysis is defined as using no or very little hazardous chemicals, getting rid of trash, and using less energy [42]. The Eco-Scale approach has been examined in order to assess the recommended spectroscopic method’s greenness [43]. The Eco-scale is a simple technique that can be applied in quality control lab operations. The following formula (analytical Eco-Scale score = 100 - total penalty) [44, 45] is used to calculate the penalty point number for each of the procedure’s given criteria, such as the amount of chemicals utilized, personnel risks, waste products, and consumption. An analysis approach is deemed green if the score is higher than 75. The created spectroscopic method’s Eco-Scale score was determined to be 91 as shown in supplementary data (S1), which is considered to be ecologically benign. The environmental friendliness of an analytical process, from sample collection to final analysis, can be evaluated using a different trend known as GAPI. The GAPI tool uses a pictogram with three color levels—green, yellow, and red—to assess how green each step of the analytical process is [46, 47]. Using the GAPI measure, the recommended method identified 6 yellow, 6 green, and 3 red zones. These areas have to do with the volume of solvent utilized and toxicity. Therefore, the recommended course of action has little effect on the environment, see Fig. 6. AGREE is the most recent metric in use. The AGREE metric’s submitted criteria are adaptable and can have varied weights. The 12 significant principles are used as a source of design inspiration. There are twelve input variables, each with a 0–1 grade [48, 49]. The final evaluation outcome is the sum of the assessment findings for each concept. The end product is a clock-like graph that has a color representation in the middle along with the overall score. Software that creates an auto-generated graph and a report can be used to do the review. According to the AGREE review, a variety of factors influence the recommended method’s 0.75, including as the number of solvents used, the volume utilized, the amount of chemicals employed in each run, and the toxicity of the solvents to people and the environment, see Fig. 6.

The modified GAPI tool (MoGAPI) [50] and associated software constitute significant advances in greenness assessment; they were created and implemented to solve the constraints of the current GAPI criteria. The given tool provides a more accurate assessment of greenness, while the software facilitates and speeds up its use. It also combines the benefits of the analytical Eco-Scale with the virtues of the commonly known GAPI metric, as seen in Fig. 6.

An innovative tool called ComplexMoGAPI [51] has been developed and successfully applied to assess the sustainability of analytical procedures. This update to the ComplexGAPI framework enables a thorough visual evaluation of the technique’s environmental impact and safety, as well as the assigning of a total score to each method. The assessment was performed using three coloring codes, namely green, yellow, and red, as well as a number indicating the total scoring, as shown in Fig. 6. The entire total score is 81.

### Blueness evaluation

To evaluate the practical considerations of an analytical approach, a new metric called the BAGI is presented [13, 52]. Two distinct sets of findings are produced by the BAGI metric tool: a numerical score located in the center of the pictogram and a graphic image shaped like an asteroid. The evaluation result is visually represented by the asteroid-shaped pictogram, which consists of many blue hues to indicate diverse levels of compliance (dark blue for high, blue for moderate, light blue for low, and white for non-compliance).

In the end, according to the assessment, which appears in the middle of the pictogram, the suggested approach receives an overall score of 80. BAGI evaluates ten elements to produce a pictogram and score that demonstrate the practicality and efficacy of an analytical technique (S2). Different shades of dark blue, blue, light blue, and white were used to designate high, medium, low, and no technical compliance with the given criteria, correspondingly, on an ordered blue color scale that was utilized for the final score representation. For the analytical method to be feasible, the ultimate score should be more than 60 (Fig. 6).

## Conclusion

New green-fitted spectrophotometric methods were created to measure pharmaceutical forms of DLX and AVN as well as mixes made in the lab. The first method based on second order derivatives while the second method is based on first derivative Dual-wavelength. The methods are novel, environmentally friendly, and accurate. For combined green spectrophotometric drug determination, the AGREE evaluation technique, the green analytical procedure index, and the analytical eco-scale were used to assess the models’ level of greenness. The outcomes demonstrated that according to the authorized green metric values that are the procedures outlined complied with and met the environmental friendliness requirements. The outcomes verified that the created models did not affect the surroundings or the analysts’ overall health. This method can be used for routine quality control tests to directly determine DLX and AVN individually or simultaneously in a binary combination with their excipients in the mixture without any prior separation. The procedure was analytically evaluated according to the International Council of Harmonization.

## Electronic supplementary material

Below is the link to the electronic supplementary material.

Supplementary Material 1

## Data Availability

Data will be made available from the corresponding author on request.
