# Supplementary material for: Novel spectrophotometric methods for concurrent assessment of duloxetine and avanafil in their binary mixture using derivative spectroscopy: greenness-blueness evaluation
Source: BMC Chem. 2025 Apr 2;19(1):87. doi: 10.1186/s13065-025-01450-0 (PMC11966798; doi:10.1186/s13065-025-01450-0)
Supplement: Supplementary file 1 — Supplementary Material 1 [file 13065_2025_1450_MOESM1_ESM.docx]

**S 1.** Evaluation of the greenness of the proposed method using the Eco scale score method.

| **Parameters** |  | **Penalty** **points** |
| --- | --- | --- |
| Reagents | Methanol | 6 |
| Instrument |  |  |
| Energy consumption | (Less than 0.1 kWh per sample) | 0 |
| Occupational hazard | (Analytical process hermitization) | 0 |
| Waste | (10 mL) | 3 |
| Total penalty points |  | 9 |
| analytical eco-scale total score |  | 91 |

^a^ If the score is greater than 75, it represents excellent green analysis. If the score is greater than 50, it represents an acceptable green analysis. If the score is less than 50, it represents inadequate green analysis.

**S2.** The 10 variables that were used to assess the suggested approach using the BAGI.

| **Parameter** | **Rating** | **Remarks** |
| --- | --- | --- |
| 1. Type of Analysis | moderately blue | Method is categorized as quantitative |
| 1. Multi-Analyte Procedure | light blue | It determines two components |
| 1. Analytical Technique Used | moderate blue | Spectrophotometric equipment is an instrument that is easily accessible in most labs |
| 1. Simultaneous Sample Preparation | light blue | The suggested method's simultaneous preparation's ease of use and time-saving nature. |
| 1. Sample Preparation | moderately blue | Sample preparation is simple and inexpensive |
| 1. Samples Per Hour | dark blue | High number of samples |
| 1. Availability of Reagents | dark blue | There are no derivative reagents—common reagents that are available commercially |
| 1. Preconcentration | dark blue | It doesn't require preconcentration |
| 1. Automation of Device | White | Procedures with manual devices |
| 1. Amount of Samples | moderate blue | The sample volume is small and direct spectrophotometric technique |
